# The magnitude of MDR carbapenemase-producing Enterobacteriaceae isolates and associated factors among hospitalized patients of Northeast Ethiopia

**DOI:** 10.1093/jacamr/dlaf080

**Published:** 2025-05-22

**Authors:** Assefa Sisay, Chalie Mulugeta

**Affiliations:** Department of Medical Laboratory College of Health Science, Woldia University, Woldia, Ethiopia; School of Midwifery, College of Health Sciences, Woldia University, Woldia, Ethiopia

## Abstract

**Background:**

Currently, carbapenemase-producing Enterobacteriaceae (CPE) are becoming a global public health threat. Infections caused by these bacteria limit treatment options and are associated with high morbidity and mortality. This study aimed to assess the prevalence of CPE and identify associated risk factors.

**Methods:**

A hospital-based cross-sectional study was conducted from June to August 2023. Clinical samples were cultured, and species identification was performed using standard biochemical tests. Antimicrobial susceptibility testing was done, and a modified carbapenem inactivation method was employed to confirm carbapenemase production. Data were entered using Epi Data and analysed with SPSS.

**Results:**

From a total of 143 isolates, the most commonly identified species were *Escherichia coli* (62 isolates, 43.4%) and *Klebsiella pneumoniae* (39 isolates, 27.3%). The highest level of resistance was against ampicillin (138 isolates, 96.5%), whereas the lowest was observed with meropenem (19 isolates, 13.3%). Overall, 123 isolates (86.0%) were classified as MDR. The prevalence of CPE and carbapenem-resistant Enterobacteriaceae (CRE) was 5.7% and 8.1%, respectively. *K. pneumoniae* and *E. coli* were the most common carbapenemase producers. Chronic underlying disease, consuming raw vegetables, and lack of regular hand-washing habits before meals showed adjusted odds ratios of 7.9 (95% CI 1.9–31.5), 11 (95% CI 3.4–40) and 8.0 (95% CI 1.7–85), respectively, showing a significant association.

**Conclusions:**

The high prevalence of CPE underscores the need for urgent infection control measures. Implementing antimicrobial stewardship, strengthening infection control measures, and further molecular studies are vital to combating this problem.

## Introduction

Antimicrobial resistance (AMR) is recognized by the WHO as a serious public health issue that requires immediate attention.^1^ β-Lactam antibiotics are commonly used to treat infections caused by MDR Enterobacteriaceae. However, these bacteria can hydrolyse certain β-lactam antibiotics due to the production of ESBLs and carbapenemases.^2^ Carbapenems are considered critically important antimicrobials for human health and are classified in the ‘Watch group’ of the WHO's Access, Watch, Reserve categorization due to their high resistance potential.^3^ The emergence of Gram-negative bacteria resistant to carbapenems is a growing global threat, with alarming increases in such cases.^4^ Carbapenem-resistant infections result in longer hospital stays, higher healthcare costs, and greater morbidity and mortality rates compared with infections caused by carbapenem-susceptible bacteria.^5^ Nearly 30 years after the initial discovery of carbapenemases in Gram-negative bacteria, they remain a significant public health concern, with carbapenemase-encoding genes now prevalent worldwide.^6^

The primary mechanisms driving carbapenem resistance include overproduction by carbapenemase genes, reduced drug permeability, modifications to PBPs and the overproduction of β-lactamases with low affinity for carbapenems.^7^ Key carbapenemase enzymes are *Klebsiella pneumoniae* carbapenemase (KPC), oxacillinase (OXA), Verona integron-encoded metallo-β-lactamase (VIM), imipenemase (IMP) and New Delhi metallo-β-lactamase (NDM).^8^ The rise of carbapenem-resistant Enterobacteriaceae (CRE) presents a major challenge in healthcare, as there are currently no viable alternative antibiotic classes to effectively treat these infections, leaving limited treatment options.^9^ Carbapenem resistance is a major public health threat, as carbapenems are often considered the last line of defence against bacteria resistant to most other antibiotics.^10^ Carbapenemase-producing bacteria are especially difficult to treat because they can break down all β-lactam antibiotics, including carbapenems, making these treatments ineffective.^11^ Additionally, the incidence of CRE has been steadily rising, although this problem remains underreported and insufficiently documented.^12^

By 2050, drug-resistant infections could claim 10 million lives annually and result in a cumulative economic loss of 100 trillion USD if proactive measures are not taken to curb their rise.^13^ Risk factors for CRE infections include a history of CRE infection, the use of urinary catheters, administration of broad-spectrum antibiotics, patient comorbidities and hospital-related factors.^14^ In sub-Saharan Africa, the limited capacity for antibiotic resistance detection and surveillance is compounded by poor hygiene, lack of access to clean water, civil conflicts, weak infection control strategies and a rising number of immunocompromised individuals.^15^ The extent of the public health threat posed by CPE in Ethiopia remains largely unknown. Moreover, to the best of our knowledge, most microbiology laboratories in Ethiopia do not perform CPE detection tests for monitoring or infection control, in addition to diagnostic testing. Therefore, this study aimed to enhance understanding and assess the prevalence of CPE, along with the associated risk factors in hospitalized patients.

## Materials and methods

### Study design, period and population

The study was conducted at Woldia Comprehensive Specialized Hospital, and Tefera Hailu Memorial Hospital, in Amhara Region, Northeast Ethiopia. These hospitals provide services including paediatrics, emergency, surgical, medical, gynaecology, psychiatry, ophthalmology, ART services, neonatal ICU, microbiological laboratories, viral load and other healthcare services.

A hospital-based cross-sectional study was conducted from June to August 2023 among patients admitted to the medical, surgical, obstetrics, gynaecology and paediatric wards or ICU for at least 48 h and who had clinical evidence of bacterial infection.

### Sample size and sampling technique

A single population proportion formula was used to calculate the sample size, assuming a 95% confidence level, 5% margin of error and a 50% prevalence of bacterial infections in hospitalized patients. The final sample size was 384. Proportional allocation was used to determine the number of study participants recruited for the study. The study participants were consecutively included until the required sample size was achieved from each ward according to their sample size allocated proportionally.

### Data collection

Socio-demographic and clinical data of study participants were collected using a structured questionnaire. For participants who couldn’t read and write, the information sheet was read to them, and a witness signed it before data collection. All the data were collected and recorded using a trained data collector.

### Clinical specimen collection and processing

Samples were collected depending on the type of infection suspected in a patient, like a urine sample from a urinary tract infection (UTI) or a blood sample for a bloodstream infection (BSI). Most patients were diagnosed with a BSI or UTI. Based on proportional allocation, stool, wound, sputum, urine and blood specimens were taken.

#### Blood sample collection

We collected aseptically 10, 5 and 2 mL blood samples from adults, paediatric patients and neonates, respectively, and added them to tryptic soy broth (TSB; Oxoid, Ltd), in two bottles for each patient, and incubated them at 37°C.^16^ Growth was followed for 7 days; if growth was observed the specimen was subcultured to blood agar (HiMedia, India) and MacConkey agar (HiMedia, India) plates. When no visible growth was detected, the TSB tubes were reported as negative.

#### Urine samples

We collected 10 mL of fresh midstream urine using a sterile, wide-mouthed, leak-proof container. Then, 1 microlitre of a well-mixed urine sample was inoculated and incubated using a cysteine lactose electrolyte-deficient medium (CLED). Colonies were counted to check for the presence of significant growth, and those yielding bacterial growth of ≥10^5^ cfu/mL were considered indicative of significant bacteriuria.^17^ The colonies from CLED agar were subcultured in MacConkey agar.

#### Sputum samples

Samples consisting of about 2 mL of purulent sputum were collected using sterile, clean, wide-necked, leak-proof containers and Gram staining was performed. Samples with fewer than 10 polymorphonuclear neutrophils per epithelial cell were considered contaminated by saliva and rejected. Sputum specimens of good quality were inoculated into MacConkey agar.^16^

#### Stool samples

Two grams of fecal specimen were collected. The sample was transported and incubated in MacConkey agar at 35°C–37°C for 24 h. From patients suspected of *Salmonella* or *Shigella* spp. infections the sample was cultured on xylose lysine deoxycholate (XLD) agar.

#### Wound samples

Using a sterile cotton swab or syringe, purulent exudates, pus and discharges were aseptically extracted from the depths of the lesion. The cotton swab was immersed in a tube containing brain heart infusion transport medium. After a 24 h incubation period, the brain-heart infusion culture was subcultured onto MacConkey agar.

### Identification of bacterial isolates

All isolates were preliminarily screened by colony morphology, pigment production and Gram staining techniques. Further identification of the isolates was performed based on relevant biochemical tests.^17^ Gram-negative bacteria were identified based on indole production, citrate utilization, urease tests, H_2_S production and carbohydrate fermentation on kligler iron agar (KIA), motility tests and lysine decarboxylase (LDC) tests.

### Antimicrobial susceptibility testing

Antimicrobial susceptibility testing was performed by the Kirby–Bauer disc diffusion method as per CLSI guidelines. Bacterial inoculates were prepared by suspending the freshly grown bacteria in 3–5 mL normal saline and turbidity was adjusted to 0.5 McFarland standard. A sterile cotton swab was dipped and rotated several times and was pressed against the wall of the test tube. It was then swabbed over the entire surface of the Mueller–Hinton agar (HiMedia, India) and antimicrobial discs were applied to the plate. Drug susceptibility testing of all Enterobacteriaceae was performed using the disc diffusion method against ampicillin (10 μg), cefoxitin (30 μg), gentamicin (10 μg), ciprofloxacin (5 μg), trimethoprim/sulfamethoxazole (1.25/23.75 μg), imipenem (10 μg), meropenem (10 μg), amoxicillin/clavulanate (20/10 μg), cefotaxime (30 μg), ceftazidime (30 μg), ceftriaxone (30 μg), tetracycline (30 μg), cefepime (30 μg) and chloramphenicol (30 μg). The diameters of the zones of inhibition were measured using a ruler and interpreted according to CLSI standards.^18^ Bacteria that are resistant to three or more antimicrobial agents belonging to different chemical classes are considered as MDR bacteria.^19^

### Screening for carbapenemases

Enterobacteriaceae exhibiting resistance to imipenem or meropenem, or a zone of inhibition of less than 19 mm for either drug, were regarded as producing a carbapenemase.^19^

### Phenotypic confirmation of carbapenemase production

Suspected carbapenemase-producing isolates were confirmed by the modified carbapenem inactivation method (mCIM) as recommended by the CLSI.^18^ To assess carbapenemase production in bacterial isolates, a suspension of the tested isolate was prepared in 2 mL of TSB, followed by the addition of a 10 μg meropenem disc. The mixture was incubated for 4 h at 35°C. After incubation, the meropenem disc was transferred onto a Mueller–Hinton agar plate previously inoculated with a suspension of *Escherichia coli* ATCC 25922 adjusted to 0.5 McFarland standard. The plate was incubated overnight (18–24 h) at 37°C. Isolates producing a zone of inhibition of 6–15 mm, showing pinpoint colonies within a 16–18 mm zone, or lacking inhibition of the meropenem-susceptible *E. coli* ATCC 25922 were identified as carbapenemase producers.^19^

### Data and laboratory quality control

The questionnaires were reviewed for completeness both during and after data collection. From specimen collection through to final bacterial identification and data management, all procedures adhered strictly to the standard operating protocols for isolate collection and laboratory analysis. The sterility of the prepared culture media was verified by incubating 5% of the prepared media, and Petri dishes were visually inspected for cracks, haemolysis and signs of contamination. The ability of the prepared media to support the growth of organisms was checked by inoculating an ATCC control strain including *E. coli* (ATCC 25922).^18^ For carbapenemase detection, BAA1705 control strain as positive control and BAA1706 as negative control were used.^20^

### Data management and analysis

Data were checked and entered using EpiData software, version 3.1, and exported to SPSS version 25 for analysis (IBM Corp., Armonk, NY, USA). Descriptive statistics were applied to summarize the relevant variables. Binary logistic regression was employed to assess the associations between dependent and independent variables. Variables with a *P* value  ≤ 0.2 in the bivariate logistic regression analysis were further included in a multivariate logistic regression for final confirmation of statistical significance. A *P* value <0.05 was considered statistically significant.

## Results

### Socio-demographic characteristics of study participants

A total of 384 study participants were presumed to have bacterial infections. Of these, the majority were males 203 (52.8%) and resided in rural areas 218 (56.8%). In this study 155 (40.4%) study participants were in the age category 30–44 years (Table 1).

**Table 1.** Socio-demographic characteristics of study participants at selected hospitals in Northeast Ethiopia (*N* = 384)

Socio-demographic characteristics	Categories	*n* (%)
Sex	Male	181 (47.2)
	Female	203 (52.8)
Age categories, y	<14	55 (14.3)
	14–29	63 (16.4)
	30–44	155 (40.4)
	45–59	81 (21.9)
	>60	30 (7.8)
Place of residence	Rural	218 (56.8)
	Urban	166 (43.2)
Occupational status	Government employee	22 (5.7)
	Merchant	40 (10.4)
	Housewife	55 (24.7)
	Day labourer	22 (5.7)
	Farmer	109 (28.4)
	Other	26 (6.8)
Hand-washing habit before meal	Yes	319 (83.1)
	No	65 (16.9)
Habit of eating uncooked vegetables	Yes	48 (12.5)
	No	336 (87.5)
Habit of eating uncooked animal products	Yes	23 (6.0)
	No	361 (94.0)

### Clinical profile of the study participants

As shown in Table 2, the study participants’ clinical profiles were recorded. Of the 384 study participants, 86 (22.4%) had a previous history of antibiotic use over the last 3 months and 62 (16.1) had a chronic underlying disease.

**Table 2.** Distribution of clinical profiled of the study participants at selected hospitals of Northeast Ethiopia

Variables	Categories	Frequency, *n* (%)
History of antibiotic use for last 3 mo	Yes	86 (22.4)
	No	298 (77.6)
History of hospitalization in the last 3 mo	Yes	97 (25.3)
	No	287 (74.7)
Previous history of ICU stays	Yes	5 (1.3)
	No	379 (98.7)
History of invasive procedure	Yes	21 (5.5)
	No	363 (94.5)
Chronic underlying disease	Yes	62 (16.1)
	No	322 (83.9)
Number of beds per room	3–5	205 (53.4)
	6–8	179 (46.6)

### Distribution of bacterial isolates and carbapenem resistance

A total of 143 Enterobacteriaceae was isolated from clinical samples. Among these, the most frequent isolates were *E. coli* (62 isolates, 43.4%) followed by *K. pneumoniae* (39 isolates, 27.3%). Of the 143 isolates, most were isolated from urine samples (*n* = 53) followed by blood samples (*n* = 41). High blood and culture infection rates indicate bacteraemia, which can result in sepsis, a potentially fatal illness that, if left untreated, can cause organ failure and septic shock. Furthermore, recurring or severe infections in urine samples are indicative of a complex urinary tract infection, which can develop into urosepsis, a potentially lethal systemic reaction to infection (Table 3).

**Table 3.** Prevalence of carbapenem-resistant Enterobacteriaceae (CRE) and carbapenemase-producing Enterobacteriaceae (CPE) of isolates against clinical specimen types

Organism	Clinical sample	Carbapenem resistance, *n* (%)		Carbapenemase production, *n* (%)		Total, *n* (%)
		Positive	Negative	Positive	Negative	
*Escherichia coli*	Blood	4 (1.0)	13 (3.4)	3 (0.8)	14 (3.6)	17 (4.4)
	Urine	3 (0.8)	22 (5.7)	2 (0.5)	23 (6.0)	25 (6.5)
	Wound	3 (0.8)	7 (1.8)	2 (0.5)	8 (2.1)	10 (2.6)
	Sputum	—	2 (0.5)	—	2 (0.5)	2 (0.5)
	Stool	—	8 (2.1)	—	8 (2.1)	8 (2.1)
*Klebsiella pneumoniae*	Blood	4 (1.0)	10 (2.6)	3 (0.8)	11 (2.9)	14 (3.6)
	Stool	2 (0.5)	1 (0.3)	1 (0.3)	2 (0.5)	3 (0.8)
	Urine	4 (1.0)	13 (3.4)	4 (1.0)	13 (3,4)	17 (4.4)
	Wound	—	5 (1.3)	—	5 (1.3)	5 (1.3)
*Klebsiella* spp.^a^	Blood	2 (0.5)	1 (0.3)	1 (0.3)	2 (0.5)	3 (0.8
	Urine	2 (0.5)	1 (0.3)	1 (0.3)	2 (0.5)	3 (0.8
	Stool	—	3 (0.8)	—	3 (0.8)	3 (0.8
	Wound	—	1 (0.3)	—	1 (0.3)	1 (0.3)
*Citrobacter freundii*	Wound	—	2 (0.5)	—	2 (0.5)	2 (0.5)
	Stool	—	2 (0.5)	—	2 (0.5)	2 (0.5)
*Citrobacter koseri*	Blood	2 (0.5)	4 (1.0)	2 (0.5)	4 (1.0	6 (1.6)
	Urine	2 (0.5)	1 (0.3)	1 (0.3)	2 (0.5)	3 (0.8)
*Proteus* spp.^b^	Urine	2 (0.5)	2 (0.5)	1 (0.3)	3 (0.8)	4 (1.0
	Blood	1 (0.3)	—	1 (0.3)	—	1 (0.3)
*Enterobacter aerogenes*	Wound	—	6 (1.6)	—	6 (1.6)	6 (1.6)
	Stool	—	5 (1.3)	—	5 (1.3)	5 (1.3)
*Providencia* spp.	Urine	—	1 (0.3)	—	1 (0.3)	1 (0.3)
	Sputum	—	2 (0.5)	—	2 (0.5)	2 (0.5)
Total		31 (8.1)	112 (29.2)	22 (5.7)	121 (31.5)	143 (37.2)

^a^ *Klebsiella* spp.: *K. ozaenae* and *K. oxytoca*.

^b^ *Proteus* spp.: *P. vulgaris* and *P. mirabilis*.

### Antimicrobial resistance pattern of Enterobacteriaceae

In this study, antimicrobial susceptibility tests for all the isolated Enterobacteriaceae were done against selected antibiotics. Bacterial isolates showed variable levels of resistance to different antimicrobial discs. The highest level of resistance among isolates was observed for ampicillin (138 isolates, 96.5%) followed by tetracycline (124 isolates, 86.7%), whereas the lowest resistance was observed for meropenem (19 isolates, 13.3%). *E. coli* showed a resistance rate of 96.8% to ampicillin and 93.5% to tetracycline, whereas resistance to carbapenems (imipenem and meropenem) was much lower at 12.9% and 14.5%, respectively (Table 4).

**Table 4.** Antimicrobial resistance profiles of Enterobacteriaceae isolates from selected hospitals in Northeast Ethiopia

Isolates (*n*)	Level of resistance to antibacterial agents, *n* (%)												
	AMP	FOX	GEN	CIP	SXT	AMC	MEM	IPM	CTX	CAZ	CRO	TET	CHL
*E. coli* (62)	60 (96.8)	24 (38.7)	23 (37.1)	33 (53.2)	48 (77.4)	41 (66.1)	8 (12.9)	8 (12.9)	35 (56.5)	37 (59.7)	38 (61.2)	58 (93.5)	25 (40.3)
*K. pneumoniae* (39)	39 (100)	21 (53.8)	20 (51.2)	24 (61.5)	26 (66.7)	31 (79.5)	6 (15.3)	10 (25.6)	28 (71.8)	29 (74.4)	23 (59.0)	34 (87.2)	19 (48.7)
*Klebsiella* spp.^a^ (10)	10 (100)	6 (60.0)	7 (70.0)	6 (60.0)	7 (70)	9 (90.0)	2 (20.0)	3 (30.0)	7 (70.0)	6 (60.0)	5 (50.0)	8 (80.0)	4 (40.0)
*C. freundii* (4)	3 (75)	2 (50.0)	1 (25.0)	2 (50.0)	3 (75)	3 (75.0)	0 (0)	0 (0.00)	3 (75.0)	3 (75.0)	1 (25.0)	3 (75.0)	3 (75.0)
*C. diversus* (9)	9 (100)	5 (55.6)	3 (33.3)	4 (44.4)	5 (55.6)	6 (66.7)	2 (22.2)	2 (22.2)	6 (66.7)	6 (66.7)	4 (44.4)	8 (88.9)	6 (66.7)
*E. aerogenes* (11)	10 (90.9)	8 (72.7)	5 (45.5)	5 (45.5)	8 (72.7)	8 (72.7)	0 (0.00)	0 (0.00)	5 (45.5)	6 (54.5)	4 (36.4)	7 (100)	6 (54.5)
*Providencia* spp. (3)	3 (100)	1 (33.3)	0 (0.0)	0 (0.0)	2 (66.7)	2 (66.7)	0 (0.0)	0 (0.0)	1 (33.3)	0 (0.0)	1 (33.3)	2 (66.7)	1 (33.3)
*Proteus* spp.^b^ (5)	5 (100)	3 (60)	2 (40)	1 (20.0)	2 (40)	4 (80)	1(20)	2 (40)	3 (60.0)	2 (40)	2 (40)	4 (90)	2 (40.0)
Total (143)	138 (96.5)	70 (49.0)	61 (42.7)	75 (52.4)	101 (70.6)	107 (74.8)	19 (14.0)	25 (17.5)	88 (61.5)	89 (62.2)	78 (54.5)	124 (86.7)	66 (46.2)

AMC, amoxicillin/clavulanate; AMP, ampicillin; CAZ, ceftazidime; CHL, chloramphenicol; CIP, ciprofloxacin; COT, cotrimoxazole; CRO, ceftriaxone; CTX, cefotaxime; FOX, cefoxitin; GEN, gentamicin; IPM, imipenem; MEM, meropenem; SXT, trimethoprim - sulfamethoxazole; TET, tetracycline.

^a^ *Klebsiella* spp.: *K. ozaenae* and *K. oxytoca*.

^b^ *Proteus* spp.: *P. vulgaris* and *P. mirabilis*.

### MDR pattern of Enterobacteriaceae

Of all the bacterial isolates only two (1.4%) *E. coli* isolates were sensitive to all selected antibiotics. The overall MDR rate (bacteria resistant to three or more different classes of drugs) of isolated bacteria was 123 isolates (86.0%). The highest rate of MDR was seen in *K. pneumoniae* and *E. coli*, with observed resistnce rates of 37 (94.9%) and 55 (88.7%) isolates, respectively (Table 5).

**Table 5.** MDR patterns of Enterobacteriaceae isolates at selected hospitals in Northeast Ethiopia

Isolates (*n*)	Level of antibiotic resistance,^a^ *n* (%)								
	R0, *n* (%)	R1, *n* (%)	R2, *n* (%)	R3, *n* (%)	R4, *n* (%)	R5, *n* (%)	R6, *n* (%)	≥R7, *n* (%)	Total MDR
*E. coli* (62)	1(1.6)	2 (3.2)	4 (6.4)	3 (4.8)	6 (9.7)	7 (11.3)	9 (14.5)	30 (48.4)	55 (88.7)
*K. pneumonia* (39)	0 (0.0)	0 (0.0)	2 (5.1)	3 (7.7)	3 (7.7)	5 (12.8)	7 (17.9)	19 (48.7)	37 (94.9)
*Enterobacter* spp. (11)	0 (0.0)	1 (9.1)	3 (27.3)	1 (9.1)	1 (9.1)	1 (9.1)	2 (18.2)	2 (18.2)	7 (63.6)
*Klebsiella* spp. (10)	1 (10.0)	1 (10.0)	1 (10.0)	0 (0.0)	2 (20.0)	1 (10.0)	0 (0.0)	4 (40.0)	7 (70.0)
*C. diversus* (9)	0 (0.0)	1 (11.1)	1 (11.1)	0 (0.0)	3 (33.3)	1 (11.1)	0 (0.0)	3 (33.3)	7 (77.8)
*C. freundii* (4)	0 (0.0)	0 (0.0)	1 (25.0)	0 (0.0)	1 (25.0)	0 (0.0)	2 (50.0)	0 (0.0)	3 (75.0)
*Providencia* spp. (3)	0 (0.0)	1 (33.3)	0 (0.0)	0 (0.0)	0 (0.0)	1 (33.3)	0 (0.0)	1 (33.3)	2 (66.7)
*Proteus* spp. (5)	0 (0.0)	0 (0.0)	0 (0.0)	1 (20.0)	1 (20.0)	0 (0.0)	1 (20.0)	2 (60.0)	5 (100)
Total (*n* = 143)	2 (1.4)	6 (4.2)	12 (8.4)	8 (5.6)	17 (11.9)	16 (11.2)	21 (14.7)	61 (42.7)	123 (86.0)

^a^R0, resistance to no antibiotics; R1–7, resistance to one, two, three, four, five, six and seven antibiotics, respectively; MDR, resistance to three or more antibiotics from different classes.

### Prevalence of carbapenemase producing and carbapenem resistant Enterobacteriaceae

**Figure 1.**
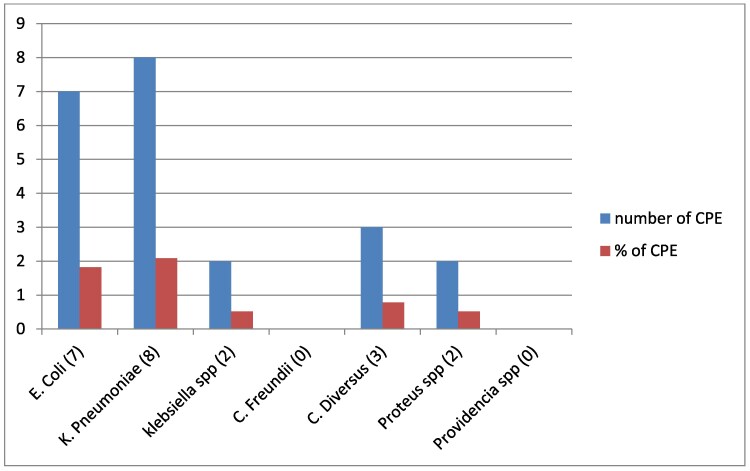
Distribution of carbapenemase-producing Enterobacteriaceae isolates from selected hospitals of North-East Ethiopia.

The prevalence of CPE and CRE was 5.7% (95% CI 3.3%–8.1%) and 8.1% (95% CI 5.2%–11.0%). These figures highlight the dominance of *K. pneumoniae* and *E. coli* among carbapenemase-producing bacteria, indicating that these two species are the primary contributors to carbapenem resistance in the studied population (Figure 1).

### Associated risk factors for acquisition of CPE infection

In logistic bivariate regression different variables were assessed. Variables with *P* ≤ 0.2 were further analysed using multivariable logistic regression. Lack of hand-washing habit before meals, the presence of chronic underlying disease, and the habit of eating raw vegetables showed statistically significant associations for the acquisition of CPE. Patients with chronic underlying disease had 7.9 times higher odds of acquiring CPE (adjusted OR = 7.9; 95% CI 1.9–31.5) compared with those without underlying conditions (Table 6).

**Table 6.** Associated risk factors for acquisition of CPE infection using bivariate and multivariable analysis from selected hospitals in Northeast Ethiopia

Variables	Category	CPE production		Bivariate analysis		Multivariable analysis	
		Positive, *n* (%)	Negative, *n* (%)	COR (95% CI)	*P* value	AOR (95% CI)	*P* value
Lack of hand-washing habit before meal	Yes	20 (6.3)	299 (93.7)	Ref			
	No	2 (3.1)	63 (96.9)	5.7 (0.7–44)	0.093	8.0 (1.7–85)	0.009*
Habit of eating uncooked vegetables	Yes	14 (29.2)	34 (70.8)	14 (5.3–38)	0.07	11 (3.4–40)	0.000*
	No	8 (2.4)	328 (97.4)	Ref			
Previous history of invasive procedure	Yes	7 (33.3)	14 (66.7)	4.8 (1.6–14)	0.004	0.8 (0.1–4.5)	0.819
	No	15 (4.1)	348 (95.9)	Ref			
Chronic underlying disease	Yes	12 (19.4)	50 (80.6)	6 (2.3–15.2)	0.001	7.9 (1.9–31.5)	0.004*
	No	10 (3.1)	312 (96.9)	Ref			
History of hospitalization	Yes	15 (15.5)	82 (84.5)	3.2 (1.1–8.2)	0.009	2.2 (0.6–8.1)	0.207
	No	7 (2.4)	280 (97.6)	Ref			
Number of beds per room	3–5	9 (4.4)	196 (95.6)	Ref			
	6–8	13 (7.3)	166 (92.7)	5.3 (1.8–15)	0.003	11(1.7–75)	0.11

AOR, adjusted odds ratio; COR, crude odds ratio; Ref, reference.

^∗^Denotes a statistically significant association between the variable and CPE prevalence (*P* ≤ 0.05).

## Discussion

The Enterobacteriaceae are adept at sharing genetic information and developing antibiotic resistance because they often acquire genes from a common pool.^21^ The emergence of carbapenemases in these bacteria is particularly concerning as they provide a much more effective and reliable mechanism of resistance to carbapenems than the combination of ESBL production and reduced permeability. Strains that produce carbapenemases are typically highly drug-resistant, as other resistance genes are often located on the same mobile genetic elements.^22^ Patients in African hospitals are at increased risk of acquiring these difficult-to-treat bacterial infections due to years of limited access to appropriate antibiotic therapies and insufficient infection control measures.^23^

In this study, a total of 143 Enterobacteriaceae isolates were obtained from clinical samples, resulting in an overall prevalence of 37.2%. This finding aligns with studies conducted in Ethiopia,^24,25^ Nigeria^26^ and India.^27^ However, this is higher than those reported in other studies from Ethiopia^28^ and Nepal.^29^ Conversely, a high percentage of infections has been reported in Nigeria^30^ and India.^31^ The observed variations may be attributed to geographical differences, variations in study methodologies, and the effectiveness of infection control programmes, and differences in patient exposure to antibiotics, which plays a significant role in the development of drug resistance.

In this study the predominant isolate was *E. coli*, which constituted 43.4% of the isolates, followed by *K. pneumoniae* at 27.3%. The results of this study regarding the predominant causes of Enterobacteriaceae infections were consistent with previous research from Ethiopia,^24^ Nigeria,^30^ Nepal^29^ and Belgium.^32^ However, these results differ from findings in other studies conducted in Ethiopia,^25,28^ Korea^33^ and Italy.^34^ This might be because these bacteria exhibit a variety of virulence factors that enhance their capacity to colonize, invade and evade the host’s immune system. Different pathogenic strains of *E. coli* are specifically adapted to various types of infections^35^ whereas *K. pneumoniae* produces capsular polysaccharides that protect it from phagocytosis and complement-mediated destruction.^36^

Since the first strain of CRE was identified in the 1980s, it has rapidly spread across the globe. Epidemiological studies suggest that certain types of carbapenemases are more common in specific regions.^37^ The emergence of CRE has become a significant public health threat, with its prevalence increasing 4-fold over the past decade, particularly among *K. pneumoniae* and *E. coli*, as highlighted in global antibiotic resistance reports.^38^ In the present study, the prevalence of CRE was 8.1%, which aligns with a study among hospitalized children in Togo,^39^ and studies from South Africa,^40^ Nigeria,^41^ New York City^42^ and Kuwait.^43^ However, it was lower than the finding of a systematic review and meta-analysis conducted in Africa,^44^ of results from a study conducted across 12 African and Asian countries,^45^ of findings from Nigeria,^30^ and of other studies from Nigeria,^46^ France^47^ and Korea.^48^ The variations in CRE prevalence and antibiotic susceptibility across different countries may be attributed to geographical differences, inadequate infection control practices in healthcare settings, and misuse, overuse and inappropriate use of antibacterials. Other factors include the availability of resources, including diagnostic facilities and trained healthcare personnel, and the persistence of CRE in the environment, equipment and hospital surfaces.^42^

Currently CPE has emerged as a significant global health issue, largely impacting patient outcomes and creating substantial therapeutic challenges. The continual emergence of carbapenemases presents diagnostic difficulties for clinical microbiology laboratories.^49^ Epidemiological data reveal that the predominant mechanism of carbapenem resistance worldwide is the production of carbapenemases, reflecting variations in resistance mechanisms.^50^ Our study revealed the overall prevalence of CPE to be 5.7%, consistent with findings from a systematic review and meta-analysis conducted in Ethiopia,^51^ and others studies from Ethiopia,^52–54^ Senegal,^55^ Lebanon^56^ and Dubai.^57^ However, it was higher than a study from Lebanon,^58^ and lower than reported by studies from Egypt,^59^ Korea^33^ and France.^47^ These discrepancies may be attributed to regional variability, differences in bacterial species, variations in carbapenemase detection techniques, localized antibiotic use and differing infection control practices. Notably, the majority of carbapenemase production was attributed to *K. pneumoniae*, which accounted for 20.5% of the total. Similar findings have been reported in studies conducted in Ethiopia,^25^ Italy^34^ and France.^47^ This might be because *K. pneumoniae* has a remarkable ability to acquire and disseminate carbapenemase genes, such as KPC, NDM and OXA-48-like enzymes, through horizontal gene transfer and is a significant cause of hospital-acquired infections, thriving in environments where invasive procedures and antibiotic use are prevalent.^4^

The rising incidence of infections caused by MDR Gram-negative bacteria poses a significant challenge in selecting the most appropriate empirical antibiotic for critically ill patients.^60^ Preventing the spread of MDR Gram-negative bacteria, particularly CRE, is challenging, making the implementation of multimodal infection control strategies essential to prevent outbreaks and mitigate severe outcomes.^61^ In our study, the overall MDR rate among Enterobacteriaceae was 86.0%. This finding aligns with similar research conducted in Ethiopia.^25,62^ It is, nevertheless, higher than reported in research from Ethiopia,^63^ Nepal^29^ and Indonesia,^64^ and less than that from Gondar, Ethiopia.^65^ This heterogeneity may be partly attributed to easy access to many routinely prescribed antibiotics in pharmacies,^66^ limited diagnostic and surveillance capacity in facing the challenges of implementing effective diagnostic and surveillance systems, and inadequate infection prevention and control measures in healthcare settings.^67^

Previous global studies have found that infection with CRE is associated with increased length of ICU stay, undergoing surgical procedures, and use of medical devices, specifically mechanical ventilation and central venous catheters.^68^ In this study, socio-demographic and clinical factors were analysed as risk factors for bacterial infection. The presence of chronic underlying disease, the habit of eating raw vegetables, and the habit of hand-washing before meals showed statistically significant association with CPE infection. According to this study, patients with chronic underlying disease had 7.9 times greater odds of acquiring CPE infection than patients without such disease. Our results are concordant with studies done in Arbaminch, Ethiopia,^69^ and from the USA^70^ and Korea.^48^ This could be due to individuals with underlying chronic illnesses being more frequently exposed to drugs, interacting with healthcare providers more often, and having compromised immune systems, all of which heighten their risk of bacterial infections.

A study emphasized that poor hand hygiene practices, along with low adherence to infection prevention and control measures, can facilitate the spread of hospital-acquired infections, including those caused by CPE.^71^ In this study individuals who had no habit of hand-washing before meals had 8.0 times greater odds of acquiring CPE infection than patients who had such a habit.

### Limitations

A molecular test for characterization of carbapenemase genes was not done for the isolated Enterobacteriaceae.

### Conclusions and recommendations

The rise of CRE and CPE is a growing concern in healthcare facilities. To tackle this issue, practical interventions such as implementing antimicrobial stewardship programmes, strengthening infection control measures, conducting regular surveillance and providing continuous education for healthcare workers are vital. We also recommend the integration of molecular diagnostics in routine clinical microbiology labs to identify the exact resistance mechanisms.

## Data Availability

The original data supporting the findings are available at any time upon reasonable request.
